# Cabergoline-Resistant Recurrent Macroprolactinoma During Pregnancy: A Case Report and Review of the Literature

**DOI:** 10.7759/cureus.102345

**Published:** 2026-01-26

**Authors:** Sharvani Alajpur, Vikram Jeet Singh Gill, Aislinn McCleery, Cathleen J Mullarkey-Desapio

**Affiliations:** 1 Internal Medicine, Saint Peter’s University Hospital, New Brunswick, USA; 2 Endocrinology, Diabetes and Metabolism, Saint Peter’s University Hospital, New Brunswick, USA

**Keywords:** cabergoline resistance, hyperprolactinemia, macroprolactinoma, recurrent macroprolactinoma in pregnancy, transsphenoidal pituitary surgery

## Abstract

We report a rare case of cabergoline-resistant recurrent macroprolactinoma during pregnancy. A 34-year-old woman, 17 weeks pregnant, presented with a four-week history of headache and a one-day history of visual disturbance. The medical history was significant for a macroprolactinoma with pituitary apoplexy, which was treated with an emergent pituitary resection approximately two years and four months before this presentation. At that time, postoperative prolactin levels improved but remained elevated despite gradually escalating cabergoline doses up to 6 mg per week. Our patient met the established criteria for cabergoline resistance, with persistently elevated prolactin levels despite high-dose cabergoline therapy. She was dealing with resistant hyperprolactinemia, causing infertility. The patient decided to conceive, and pre-pregnancy counseling was done by reproductive endocrinology, maternal-fetal medicine, and a genetic counselor. Before conception, magnetic resonance imaging (MRI) of the pituitary showed postoperative changes from transsphenoidal hypophysectomy and expanded partial sella, with no clear tumor, and the prolactin level was 228 ng/mL (non-pregnant normal reference range: 3-30 ng/mL). She had in vitro fertilization done, and cabergoline was discontinued on confirmation of pregnancy. On presentation, MRI of the pituitary revealed a recurrent pituitary macroadenoma (29 × 29 × 27 mm). She developed symptomatic tumor recurrence within three months of stopping cabergoline, and her prolactin level was 1,669.8 ng/mL (normal reference range in pregnancy: 10-209 ng/mL). She was treated with steroids, and cabergoline was restarted. However, the tumor continued to grow, causing symptoms necessitating repeat urgent pituitary resection in the second trimester of pregnancy. She remained on cabergoline through the rest of her pregnancy and delivered a healthy male neonate via cesarean section, done for breech presentation at 39 weeks. The postpartum, fetal, and neonatal stages were all uneventful. This case highlights the complexities of managing dopamine agonist-resistant macroprolactinomas during pregnancy and underscores the importance of individualized, multidisciplinary decision-making to optimize maternal and fetal outcomes.

## Introduction

Prolactinomas are the most common pituitary neuroendocrine tumors. First-line treatment is medical therapy with dopamine agonists (DA). Cabergoline is preferred over bromocriptine due to its higher efficacy and tolerability. Resistance to cabergoline has been reported in 10% of cases, defined as a failure to achieve normoprolactinemia and/or a less than 30% decrease in tumor diameter on maximally tolerated doses administered for at least 3-6 months. The maximally tolerated dose for cabergoline varies but is usually ≥2 mg per week [1]. Proposed mechanisms of cabergoline resistance include reduced dopamine D2 receptor expression, post-receptor signaling abnormalities, increased tumor fibrosis, and intrinsically aggressive tumor behavior.

Management of prolactinomas during pregnancy is challenging. There are a risk of tumor enlargement and a rise in prolactin levels due to lactotroph hyperplasia during pregnancy, attributed to the increased serum estrogen levels that may cause mass-effect symptoms. The risk of tumor enlargement may occur in 3% of those with microadenomas, 32% of those with macroadenomas that were not previously operated on, and 4.8% of those with macroadenomas with prior ablative treatment [2]. In women with prolactinoma, discontinuation of DA therapy at the time of pregnancy confirmation is recommended by current guidelines to minimize fetal exposure. Cessation of DA therapy may unmask tumor progression in susceptible patients, particularly those with residual or resistant disease [3,4].

We present a unique case of recurrent, cabergoline-resistant macroprolactinoma during pregnancy requiring repeat transsphenoidal surgery (TSS). To our knowledge, such cases are exceedingly uncommon. This report highlights the potential for rapid symptomatic tumor progression following the cessation of DA therapy during pregnancy and underscores the importance of close monitoring and multidisciplinary management in high-risk patients. We aim to create awareness among internists and endocrinologists about the possibility of symptomatic tumor recurrence within just three months after stopping medical therapy during pregnancy and the challenges involved in management.

## Case presentation

A 34-year-old primigravid woman at 17 weeks of gestation presented to our hospital in March 2024 with a four-week history of progressively worsening headache and a one-day history of visual disturbance.

Her medical history was notable for similar symptoms in November 2021, at which time imaging revealed a large expansile pituitary mass measuring 1.4 cm with a large fluid-filled component containing blood or protein, compression of the optic chiasm, and probable invasion of the right cavernous sinus. Serum monomeric prolactin was markedly elevated at 1,603 ng/mL (non-pregnant reference range: 3-30 ng/mL). She underwent emergent TSS due to concern for pituitary apoplexy. Surgical pathology confirmed a densely granulated lactotroph pituitary adenoma. Postoperatively, prolactin decreased to 428 ng/mL but remained significantly elevated, and cabergoline was initiated at 0.25 mg twice weekly. Despite gradual dose escalation to a maximum of 6 mg weekly, serial outpatient prolactin measurements demonstrated persistent hyperprolactinemia with an incomplete biochemical response. Evaluation for macroprolactin did not demonstrate a significant contribution to the elevated prolactin levels. In September 2022, prolactin levels remained elevated at 241.9 ng/mL while on cabergoline 1.5 mg three times weekly. A trial switch to bromocriptine resulted in further biochemical worsening, with prolactin increasing to 386 ng/mL and subsequently to 675 ng/mL, prompting the reinitiation of cabergoline in December 2022, which then led to partial improvement, with prolactin decreasing to 288.1 ng/mL by February 2023.

The patient desired pregnancy but was experiencing a hypoestrogenic state due to persistent hyperprolactinemia, resulting in infertility. Cabergoline was increased to 1.5 mg four times weekly (total weekly dose of 6 mg), which she tolerated well. At the patient's request, she established care with reproductive endocrinology. Maternal-fetal medicine counseled about the potential maternal risks of pregnancy, including adenoma growth with resultant neurologic symptoms, most notably visual impairment, as well as potential fetal risks related to DA exposure. She also underwent genetic counseling. Magnetic resonance imaging (MRI) of the pituitary performed immediately prior to conception demonstrated postoperative changes from transsphenoidal hypophysectomy, with an expanded partially empty sella and a small rind of homogeneously enhancing soft tissue along the floor of the sella turcica, felt to represent a combination of remnant pituitary tissue and postoperative changes; residual tumor could not be entirely excluded. After discussion of the risks, the patient was subsequently optimized for infertility treatment and pregnancy, and she elected to proceed with embryo transfer in December 2023. Following confirmation of pregnancy, cabergoline was discontinued, and she was monitored regularly in the outpatient setting by endocrinology and maternal-fetal medicine prior to her presentation to our hospital in March 2024.

Our patient had no family history of prolactinomas or other pituitary or endocrine tumors. Notably, she never had any galactorrhea before the postpartum period. Amenorrhea may not be fully attributable to hyperprolactinemia, as she had a hormonal intrauterine device in place prior to conception that was removed in June 2022, without the subsequent resumption of menstrual cycles.

Diagnostic assessment

**Figure 1:**
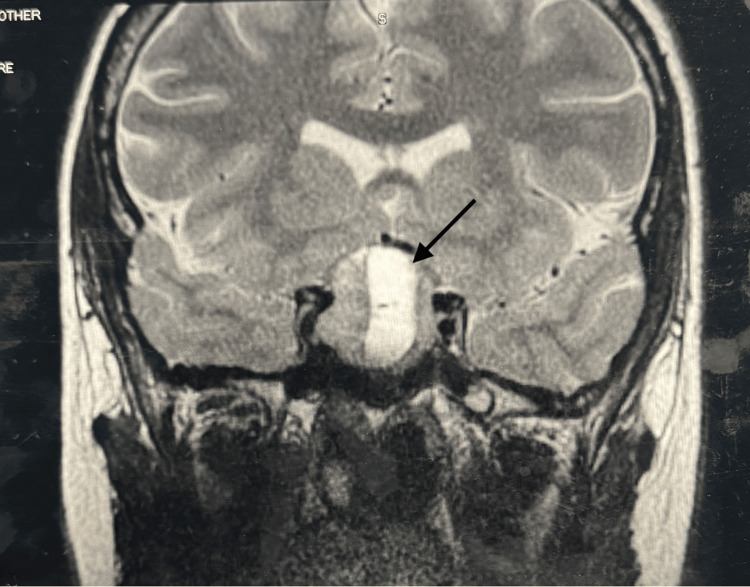
MRI of the pituitary on admission showing recurrent pituitary macroadenoma (29 × 29 × 27 mm) with central hemorrhagic component, compression of the optic chiasm, suprasellar cistern extension, and projection into the right cavernous sinus. MRI: magnetic resonance imaging

On physical examination, vital signs were normal. There were no gross visual or focal neurological deficits; however, formal visual field testing could not be performed in the inpatient setting due to a lack of equipment. The bedside confrontation visual field testing was normal. MRI of the pituitary showed a recurrent pituitary macroadenoma (29 × 29 × 27 mm) with central hemorrhagic component, compression of the optic chiasm, suprasellar cistern extension, and projection into the right cavernous sinus (Figure 1). The monomeric prolactin level was 1,669.8 ng/mL (normal reference range in pregnancy: 10-209 ng/mL), increased when compared to the pre-pregnancy prolactin level of 228 ng/mL (non-pregnant normal reference range: 3-30 ng/mL). Other pituitary hormone levels were checked as mentioned in Table 1. Follicle-stimulating hormone (FSH) and luteinizing hormone (LH) levels were low, appropriate for pregnancy. FSH was <0.7 mIU/mL, and LH was <0.2 mIU/mL. Adrenocorticotropic hormone (ACTH) levels were 12 pg/mL (normal range: 6-50 pg/mL). Thyroid-stimulating hormone (TSH) was 0.496 uIU/mL (normal range: 0.465-4.68 uIU/mL). Growth hormone (GH) levels were 0.6 ng/mL (normal range: ≤7.1 ng/mL).

**Table 1:** Patient's hormone levels showing a markedly elevated prolactin (1,669.8 ng/mL) well above the pregnancy reference range, with FSH and LH appropriately suppressed for pregnancy. ACTH, TSH, and GH are within normal limits, indicating overall preserved pituitary function. FSH: follicle-stimulating hormone; LH: luteinizing hormone; ACTH: adrenocorticotropic hormone; TSH: thyroid-stimulating hormone; GH: growth hormone

Hormone	Measured level	Reference range (pregnancy/normal)
Prolactin (monomeric)	1,669.8 ng/mL	Pregnancy: 10-209 ng/mL; pre-pregnancy: 3-30 ng/mL
FSH	<0.7 mIU/mL	Low/appropriate for pregnancy
LH	<0.2 mIU/mL	Low/appropriate for pregnancy
ACTH	12 pg/mL	6-50 pg/mL
TSH	0.496 uIU/mL	0.465-4.68 uIU/mL
GH	0.6 ng/mL	≤7.1 ng/mL

Treatment

**Figure 2:**
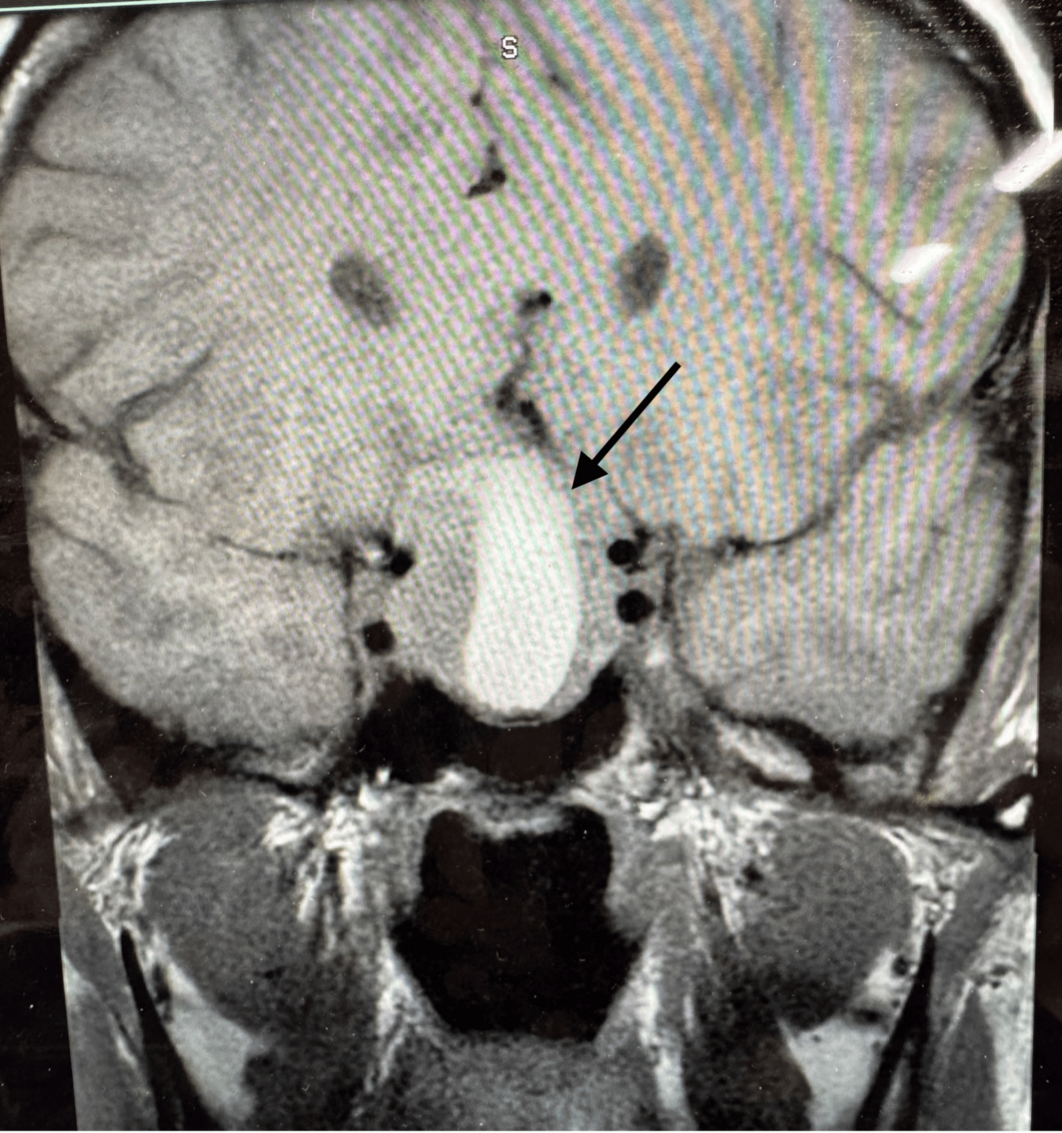
MRI of the pituitary five days after treatment showing a pituitary mass measuring 30 × 25 × 20 mm. MRI: magnetic resonance imaging

A multidisciplinary team, including an endocrinologist, a neurosurgeon, and a maternal-fetal medicine specialist, guided the treatment plan. As per the Endocrine Society Clinical Practice Guidelines, cabergoline was restarted at 1.5 mg daily to achieve rapid suppression of prolactin in the setting of acute neurologic symptoms, which she received for five days. In the setting of acute headache and visual disturbance during pregnancy, glucocorticoids (hydrocortisone 100 mg three times a day) were used as adjunctive therapy to mitigate mass effect and stabilize symptoms pending disease-directed management. Repeat imaging done five days later showed a small decrease in the size of the pituitary mass to 30 × 25 × 20 mm (Figure 2). After a transient improvement in symptoms, the patient was discharged with a prescription for cabergoline 1.5 mg, four times a week, a therapeutic dose that previously achieved tumor control and a tapering course of prednisone (prednisone 80 mg daily tapered by 10 mg every three days) with a plan for repeat TSS at a specialized neurosurgical center.

Three days after discharge, she went to the emergency department at another hospital as she developed a new visual disturbance in the inferior temporal quadrant of the right eye. Imaging then showed the pituitary macroadenoma causing significant optic chiasm compression, and the prolactin level was 2,713 ng/mL (normal reference range in pregnancy: 10-209 ng/mL). She underwent repeat urgent TSS in the second trimester of pregnancy, generally considered the safest window during pregnancy. Postoperative prolactin level was 224 ng/mL (normal reference range in pregnancy: 10-209 ng/mL), and imaging confirmed the successful decompression of the optic chiasm. The surgical pathology with immunohistochemistry for pituitary hormones and transcription factors is diagnostic of a PIT1 lineage lactotroph pituitary neuroendocrine tumor (pituitary adenoma).

Outcome and follow-up

**Figure 3:**
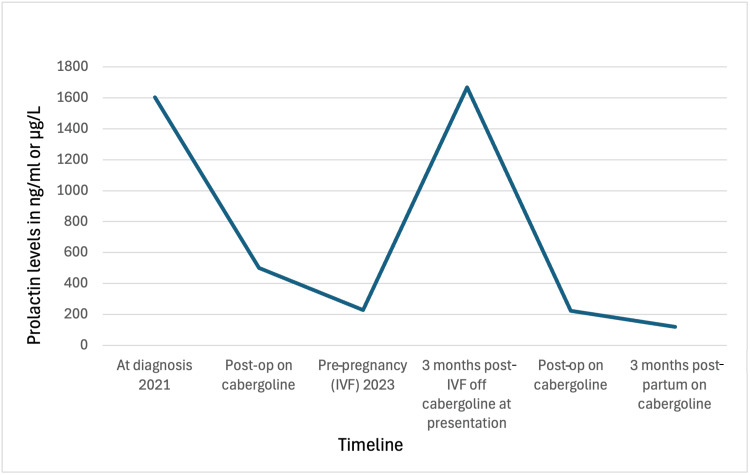
Trends in serum prolactin levels over time in relation to diagnosis, treatment, and reproductive events. Prolactin levels (ng/mL) from 2021 to 2024 are plotted against the clinical timeline. At diagnosis in 2021, prolactin was markedly elevated (1,603 ng/mL). Following TSS, levels decreased to 428 ng/mL, and while on cabergoline, prolactin level was 228 ng/mL before in vitro fertilization in 2023. Discontinuation of cabergoline during early pregnancy was associated with an increase in prolactin (1,669 ng/mL at 17 weeks), which peaked at 2,713 ng/mL prior to repeat TSS during pregnancy. Postoperatively, prolactin levels decreased but remained above the reference range (224 ng/mL). At three months postpartum, prolactin levels continued to be elevated (120 ng/mL) but lower compared to preoperative pregnancy levels, consistent with persistent biochemical disease despite ongoing dopamine agonist therapy. TSS: transsphenoidal surgery

After the surgery, she continued to be on cabergoline 0.5 mg four times a week as the prolactin levels were still elevated. She developed postoperative central hypothyroidism treated with levothyroxine 50 mcg daily and secondary adrenal insufficiency treated with hydrocortisone 10 mg in the morning and 5 mg in the evening, along with stress-dose steroids during delivery. She delivered a healthy male neonate via cesarean section at 39 weeks with no maternal or neonatal complications. The indication for cesarean section was fetal breech presentation. She had profuse galactorrhea postpartum and had no problems with breastfeeding. Breastfeeding and DA management did not influence one another. Prolactin levels at key clinical milestones are summarized in Figure 3, providing a visual overview of values over the course of her disease. She continues to have persistently elevated prolactin levels while on cabergoline and is currently being monitored at our outpatient endocrinology department. One year postpartum, total prolactin was 113 ng/mL, with a monomeric fraction of 91 ng/mL (80% of total), confirming that the elevation was predominantly biologically active. Given the timing, cessation of lactation, and ongoing cabergoline therapy, this persistent mild hyperprolactinemia is consistent with partial biochemical resistance rather than physiologic postpartum elevation. MRI of the pituitary showed no recurrence of the tumor. She also follows an ophthalmologist for regular visual field assessment. Postpartum evaluation showed excellent visual acuity with mild bilateral visual field defects.

## Discussion

The management of prolactinoma during pregnancy presents complex clinical dilemmas. In our patient, the rapid expansion of a recurrent macroadenoma with acute visual symptoms necessitated the urgent consideration of TSS, which is generally safest in the second trimester. Concurrently, the decision to restart cabergoline involved weighing the maternal benefits of tumor control against potential fetal exposure, guided by limited but reassuring safety data. Throughout this process, multidisciplinary counseling with endocrinology, neurosurgery, and maternal-fetal medicine was essential to ensure that the patient was fully informed of the risks and benefits of each intervention, including the potential need for repeat surgery or the escalation of medical therapy, allowing her to participate actively in shared decision-making.

Distinguishing true cabergoline resistance from physiological prolactin elevation following DA withdrawal is critical in clinical decision-making. Cabergoline resistance is defined by persistently elevated prolactin levels and/or lack of tumor shrinkage despite adequate, often high-dose therapy. In contrast, DA withdrawal results in a transient rise in prolactin that typically occurs within days to weeks after discontinuation, often accompanied by the resumption of menses if the patient is not pregnant. In our patient, prolactin remained elevated despite ongoing cabergoline therapy, supporting the presence of partial biochemical resistance rather than a temporary withdrawal effect.

Risk factors for cabergoline resistance

Cabergoline-resistant prolactinomas represent a clinical challenge, as DAs are first-line therapy. Resistance is more frequently observed in patients with large, invasive macroadenomas. Prior studies have identified male sex, cavernous sinus invasion, and tumor size as key risk factors for resistance, with a subset of patients requiring high-dose cabergoline without achieving biochemical control [5]. In resistant cases, surgical debulking has been shown to improve prolactin control and reduce medication requirements, supporting a multimodal, individualized treatment approach [6]. Genetic and molecular factors may contribute to DA resistance, including reduced dopamine D2 receptor expression and altered downstream signaling [7]. AIP gene mutations, associated with familial pituitary adenomas, have been linked to reduced responsiveness to DA, although their impact in sporadic prolactinomas remains uncertain [8]. Additionally, a higher proliferative index (Ki-67 ≥3%) has been associated with larger, more invasive tumors, lower rates of prolactin normalization, and increased recurrence, reflecting a more aggressive disease phenotype [9]. In our patient's case, the Ki-67 proliferation index was not reported in the pathology specimen and could not be assessed.

Several factors may have contributed to the aggressive and cabergoline-resistant behavior observed in this patient. Pathologically, the tumor was a densely granulated lactotroph adenoma, and its macroadenoma size, invasive features, and prior hemorrhagic event may have predisposed it to rapid recurrence. While no familial history, genetic testing for AIP mutations or other syndromic pituitary adenomas like MEN1 was performed, such evaluations could provide insight into intrinsic tumor aggressiveness and DA responsiveness. These clinical and potential molecular features underscore the importance of individualized monitoring and therapeutic planning in high-risk prolactinoma cases.

Prolactinoma in pregnancy

The European Society of Endocrinology recommends discontinuing DA once pregnancy is established, especially after tumor shrinkage is confirmed [3]. However, DA therapy can be continued throughout pregnancy if the adenoma is large abutting the optic chiasm, and it may be restarted later in pregnancy if there are symptoms of progressive tumor growth. In cases of symptomatic tumor enlargement, medical treatment with DA should be attempted before considering TSS. Surgical intervention for pituitary tumors during pregnancy is rare and should generally be avoided unless there is a medical failure or severe visual disturbances caused by tumor growth or apoplexy. Radiotherapy is not recommended during pregnancy due to the delayed onset of effects and potential fetal harm. Pregnant women with large macroprolactinomas or tumors near the optic chiasm should receive individualized follow-up, including clinical assessment, vision testing, and an MRI without contrast if vision worsens. Monitoring prolactin levels is not clinically helpful during pregnancy, as physiological elevations make measurements unreliable.

For future pregnancies, careful pre-conception planning is essential in patients with a history of recurrent or dopamine agonist-resistant prolactinoma (DARP). Recommended management includes achieving maximal tumor control prior to conception, ideally with prolactin normalization using the lowest effective dose of DA. Pre-conception counseling should involve a multidisciplinary team, including endocrinology, maternal-fetal medicine, and a genetic counselor, to discuss potential risks of tumor growth during pregnancy, indications for reinitiating therapy, and the timing of imaging if symptomatic. Patients should be educated on warning signs such as headache or visual changes, and individualized plans should be established to allow prompt intervention while balancing maternal and fetal safety.

Compared with the previously reported case of a young woman with recurrent invasive macroprolactinoma successfully managed with continued cabergoline throughout pregnancy, our case illustrates a more complex and aggressive disease course [10]. While both patients had large invasive tumors with prior TSS and achieved pregnancy under DA therapy, key differences include the presence of rapid tumor expansion with acute visual compromise in our patient, partial biochemical resistance to cabergoline, and the need to consider surgical intervention during pregnancy. In contrast, the comparator case demonstrated biochemical normalization, radiographic stability, and an uncomplicated pregnancy without tumor progression. These differences underscore the heterogeneity of prolactinoma behavior in pregnancy and highlight that prior surgical debulking and initial DA responsiveness do not reliably predict stability in subsequent pregnancies. Our case adds novelty by emphasizing the clinical decision-making dilemmas surrounding DA resistance, timing of surgery, and multidisciplinary counseling in a high-risk pregnancy, extending existing literature beyond cases of stable DA-responsive disease.

Second-line and emerging therapies

Treatment options for DARP are limited. Temozolomide is recommended as first-line chemotherapy for aggressive pituitary neuroendocrine tumors and has demonstrated efficacy in selected cases of DARP, particularly in tumors with low MGMT expression [11]. Somatostatin analogs, including pasireotide, have shown tumor and biochemical control in isolated aggressive cases, though broader efficacy remains unproven [12]. Experimental therapies, such as anti-estrogen agents, tyrosine kinase inhibitors, and molecular pathway-targeted approaches, have shown promise in preclinical or limited clinical settings but require further validation before routine use [13-17].

Surgical management

When medical therapy fails, TSS remains the primary treatment for resectable DARP, particularly to relieve mass effect. Tumor recurrence may occur, and radiotherapy may be considered in selected cases, although its effectiveness is limited and delayed.

## Conclusions

This case illustrates the complex challenges of managing a prolactinoma in pregnancy, particularly in the setting of cabergoline resistance and tumor recurrence with invasive features. Despite initial surgical resection and high-dose DA therapy, our patient experienced rapid regrowth of a macroadenoma after a brief discontinuation of cabergoline during pregnancy. Her course emphasizes the need for individualized pre-pregnancy counseling to discuss recurrence risks, potential complications, and the importance of multidisciplinary care. In women with a history of aggressive or resistant prolactinomas, careful monitoring of symptoms and tumor size during pregnancy is essential, and medical therapy may be continued when the anticipated benefits outweigh potential risks. Beyond individual patient care, this case underscores the broader gap in evidence regarding the safety of cabergoline in pregnancy and the limited options for treating cabergoline-resistant disease. Continued research is needed to guide management strategies, reduce recurrence risk, and improve outcomes for women of reproductive age with high-risk prolactinoma.
